# Lower Urinary Tract Symptoms (LUTSs) in Elite Female and Male Athletes: Prevalence and Impact on Performance—A Cross-Sectional Study Using the STROBE-SIIS (Sports Injury and Illness Surveillance) Reporting Guidelines

**DOI:** 10.3390/epidemiologia7020046

**Published:** 2026-04-01

**Authors:** Beth McCullough, Thomas Fallon, Neil Heron

**Affiliations:** 1 Centre for Public Health, Queen’s University Belfast, Belfast BT12 6BJ, UK; bmccullough09@qub.ac.uk (B.M.); tfallon02@qub.ac.uk (T.F.); 2 Edinburgh Sports Medicine Research Network & UK Collaborating Centre on Injury and Illness Prevention in Sport (UKCCIIS), Institute for Sport, PE and Health Sciences, University of Edinburgh, Edinburgh EH8 8AQ, UK

**Keywords:** lower urinary tract symptoms, LUTS, urinary incontinence, UI, prevalence, elite athletes

## Abstract

**Introduction:** Lower urinary tract symptoms (LUTSs), including urinary incontinence (UI), are common problems present in the general population. However, these symptoms have also been seen in young, elite-level athletes, especially females, including those who are nulliparous. This preliminary study aimed to report on the prevalence of LUTSs within an elite athletic population, including both males and females, within the UK high-performance system (the Sports Institute of Northern Ireland) and a women’s elite cycling team, while also investigating the link between LUTSs and specific training and sporting activities. **Methods:** A cross-sectional study of elite athletes in the Sports Institute of Northern Ireland (SINI) and a women’s professional cycling team, using an online questionnaire, was conducted to investigate the prevalence of LUTSs and UI and their impact on quality of life (QOL) among both male and female elite athletes. The authors used the STROBE-SIIS guidelines to produce separate electronic questionnaires for male and female athletes. This is a preliminary pilot study due to the small sample size. **Results:** Ten male athletes completed the IPPS questionnaire, reporting a median score of 5.5/35. Meanwhile, 18 female athletes completed the Athlete Female LUTS (A-FLUTS) questionnaire and reported a median score of 6/44. Female athletes had a higher prevalence of UI in the last four weeks (66.7%) compared to male athletes (20%). Of the 28 athletes, 7 were explosive/sprint athletes, and 21 were endurance athletes. Explosive/sprint athletes (71.4%) appeared to have a higher prevalence of UI in the last four weeks compared to endurance athletes (42.9%). Athletes self-managed these UI symptoms through a variety of methods, including reducing fluid intake, which could impact their athletic performance. This is a preliminary pilot study and—despite its small size—it defines a methodology and shows some important results that encourage research to be carried out on a larger sample size. **Conclusions:** The reported QOL impact and potential impact on health and athletic performance highlight the need for better management and treatment protocols, including the need to screen for urinary symptoms in the pre-season medical.

## 1. Introduction

Lower urinary tract symptoms (LUTSs), including urinary incontinence (UI), are conditions that are reportedly prevalent within the general population, especially in older individuals. A recent study showed that 81.4% of females over sixty-five had experienced LUTSs. The prevalence of LUTSs in males over sixty-five was 76.6% and 51.3% in males aged eighteen to thirty-nine [1,2]. LUTS is a collective term to describe symptoms affecting urination, which can be broadly categorised into three groups: storage, voiding and post-micturition symptoms. Storage symptoms include UI, increased urinary frequency, nocturia and urgency. Symptoms associated with voiding include hesitancy, weak stream, and terminal dribbling, whereas incomplete emptying and postmicturition dribbling are classified as postmicturition symptoms [3]. Whilst LUTSs and UI are often accepted as an inevitable ageing process in females with post-partum and post-menopausal associations, recent research has shown that these symptoms are prevalent in young, nulliparous female athletes [4]. The prevalence of UI in elite female athletes has been reported in the literature as between 5.7% and 80% [5,6]. Female athletes experience a 177-percent increased risk of developing UI when compared to sedentary women [5].

LUTSs in male elite athletes are less well understood and investigated. However, they have been reported to have a prevalence of 14.7% [7]. The higher prevalence of these symptoms within athletes is thought to be an underrepresentation, as athletes can often underreport the severity of their symptoms due to embarrassment or fear of impact on their sport [4]. This underrepresentation and lack of acceptance of symptoms, especially in female athletes, result in a lack of engagement with healthcare professionals and, therefore, a lack of appropriate management of their symptoms [7].

For both genders, LUTS management ranges from conservative methods to medical and then surgical if the previous methods prove unsuccessful. Conservative methods include limiting fluid intake, the use of pads and absorbency products, and reducing caffeine and carbonated drinks. For males, urethral milking can be effective [8]; for women experiencing pelvic organ prolapse, pelvic floor exercises are recommended. No specific management for elite athletes is mentioned in these guidelines, and this needs to be considered to ensure appropriate management of these symptoms within the unique world of elite sports [9].

Patient-reported outcome measures (PROMs) are a useful and reliable method for investigating the prevalence of LUTSs in elite athletes and allow data to be collected anonymously and remotely, which can increase the response rate [7]. The International Consultation on Incontinence Questionnaires (ICIQ) was developed to provide PROMs with a standardised, high-quality assessment of symptoms that were urinary, vaginal or bowel-related. This has been used for the past twenty-two years [10]. The ICIQ FLUTS (female lower urinary tract symptom) is a modular questionnaire developed through a controlled matched study and validated. The controlled matched study included a female population who underwent urodynamic investigation for UI symptoms. It investigates LUTSs and their impact on the patient’s quality of life. Taking around four to five minutes to complete, its 12 items have established validity, reliability, and responsiveness [11]. A-FLUTS is an adaptation of the ICIQ FLUTS questionnaire, specially adapted to athletes. Meanwhile, in males, LUTSs can be assessed using the International Prostate Symptom Score (IPPS) questionnaire, which is a valid and reliable method of data collection recommended by NICE in the United Kingdom (UK) for this purpose [8,12].

The large impact of LUTSs on quality of life, mental health and physical health has been highlighted recently [4]. Research into this topic is therefore important to form guidelines to improve the management of these symptoms and, therefore, increase the quality of life and performance of elite athletes, both male and female.

### 1.1. Normal Anatomy

Pelvic anatomy consists of the closely related bony pelvis and pelvic floor. The bony pelvis is made up of two innominate bones, each consisting of an ilium, ischium and pubis, that articulate anteriorly to form the pubic symphysis and posteriorly with the sacrum to form the sacroiliac joint (SIJ) [13].

**Figure 1:**
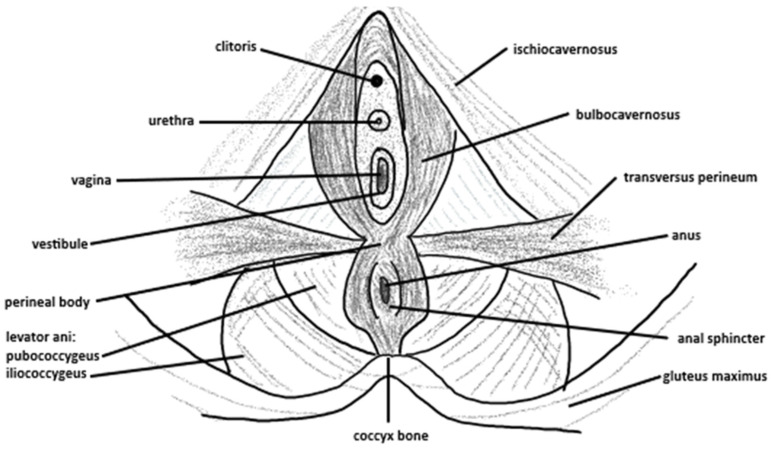
An illustration of the female pelvic floor, including bony, anatomical and muscular landmarks.

**Figure 2:**
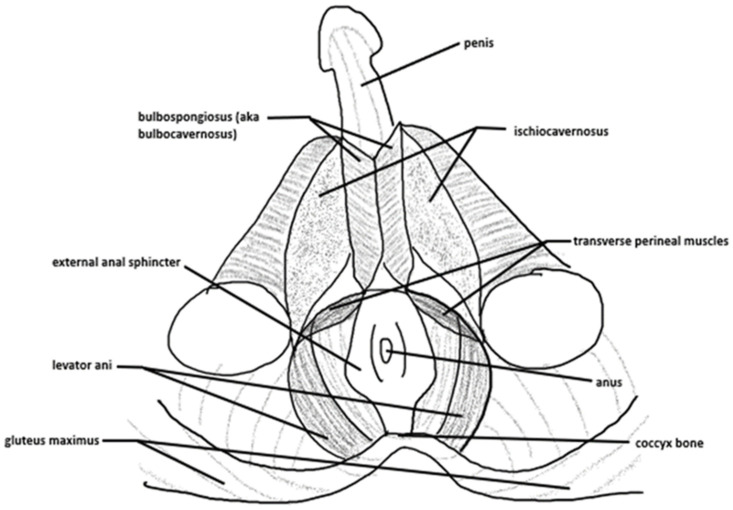
An illustration of the male pelvic floor, including bony, anatomical and muscular landmarks.

Within the bony pelvis, the pelvic floor acts as a muscular sling which aids stability and supports the organs of the pelvis, including the bladder, rectum, and uterus (females only), to prevent pelvic organ prolapse. The anus and urethral openings also form a part of the pelvic floor. In females, the vagina is also included. The pelvic floor can be divided into superficial and deep aspects, with the superficial pelvic floor consisting of the ischiocavernosus, bulbospongiosus and transverses perineal (superficial and deep in males, superficial only in females) muscles and the deep pelvic floor consisting of the levator and muscles (pubococcygeus, iliococcygeus, and puborectalis muscles) and coccygeus muscle [14]. The lateral aspect of the pubic symphysis at the posterior surface of the superior pubic ramus acts as the origin for the levator ani muscles, which insert onto the inner surface of the ischial spine [15]. An illustration of the female pelvic floor can be viewed in Figure 1, and the male pelvic floor in Figure 2.

The levator ani muscles, together with the coccygeus, form the pelvic diaphragm. These muscles always demonstrate electrophysiologic activity to maintain tone even when at rest to provide support to the pelvic contents. [16]. Between the rectum and the vagina/penis is the perineal body, which provides support for the pelvic floor and is composed of fascial tissue [4]. The pudendal nerve gives off branches that provide motor supply to the urinary and anal sphincters, general sensation to the external genitalia, and branches for orgasmic and ejaculatory function. Its tortuous nature leaves it susceptible to injury in pelvic surgery and childbirth, and it is a common source of pelvic pain and dysfunction in cyclists [17,18].

The anatomy of the pelvis differs slightly based on gender. The male pelvis is narrower and deeper than females, with the former having a lower pelvic capacity. In males, the level of the levator ani, when compared to the level of the upper edge of the pubic ramus, is lower than in females [19]. Typically, the male pelvic inlet is narrow and heart-shaped, known as “android” in shape, whereas the female pelvic inlet is wide and oval-shaped, known as “gynecoid” [20]. The levator ani muscles are better developed in females than males, possibly due to their more transverse orientation. This creates less favourable leverage of these muscles in females [21]. In both sexes, urinary continence is controlled by the detrusor, trigone, and urethral sphincter muscles [22].

### 1.2. Embryology

Appreciation of the developmental anatomy of the urethral sphincter complex and pelvic floor muscles is crucial in the understanding and treatment of urinary incontinence in both males and females [22]. In early primates, support of the pelvic organs was controlled via caudal muscle contraction, pulling the tail root against the peritoneum [23].

The embryology of the pelvic floor remains largely unclear, with new concepts still being produced. The levator ani muscles are representative of evolutionary remnants of tailed mammalians. The differences between male and female levator ani muscles become more evident by the second trimester [24].

### 1.3. Pathophysiology

UI can happen for many different reasons, including but not limited to medications, over- or underactivity of the bladder, neurological problems, pelvic organ prolapse, constipation, urinary infection, performing high-level sport and weak pelvic floor muscles. [25]. UI can be subdivided into a few different types, including stress, urge, mixed, overflow and functional incontinence. Stress incontinence involves involuntary leakage of urine on exertion, such as when coughing, sneezing, or jumping due to weakened pelvic floor muscles, increased mobility of the urethra, poor functioning of the intrinsic sphincter, or a combination of all three [26]. Urge incontinence involves a sudden need to urinate, which leaks before reaching the toilet, usually resulting from detrusor overactivity. Mixed incontinence refers to symptoms of both urge and stress incontinence, which is common in older women. Overflow incontinence refers to leakage of urine from an overdistended bladder, usually due to an obstruction or neurological condition. Functional incontinence describes the leakage of urine from a fully functional urinary system, usually due to difficulties accessing the toilet due to cognitive, neurological or mobility issues [26]. The most common type of incontinence experienced by female athletes is stress incontinence due to weakness or damage to the pelvic floor muscles during strenuous exercise [24]. Factors including training duration, intensity and frequency may impact the extent of UI in female athletes. A study of pelvic floor muscle pressure in female athletes found that training intensity and duration shared a negative association with perineal pressure, which in turn was associated with higher levels of UI [27]. There are many risk factors for UI, including increased BMI, participation in high-impact sports, eating disorders, foot flexibility, smoking, drinking carbonated drinks and altered urinary microbiome [4]. A diagnosis of Relative Energy Deficiency in Sport (RED-S) can also be a risk factor for UI in female athletes. RED-S is a syndrome due to low energy availability that results in performance and health impairments. In female athletes, RED-S will cause a reduction in oestrogen, impacting pelvic floor health due to the loss of this protective hormone and potentially causing UI [28]. Additionally, RED-S can also reduce urethral sphincter muscle strength, resulting in insufficient closure during high-impact activities and consequent involuntary leakage of urine [28].

### 1.4. Aims

The objectives of this study are to report on the prevalence of LUTSs within an elite athletic population, including both males and females, within the UK high-performance system (the Sports Institute of Northern Ireland) and a women’s elite cycling team. This study also aims to investigate the link between LUTSs and specific training and sporting activities.

## 2. Methods

This is a cross-sectional study that investigates the prevalence of LUTSs in both male and female elite athletes in the SINI and a women’s elite cycling team (World Tour level). These athletes were chosen as a convenience sample as the researcher, NH, had access to these professional athletes. The study was developed using the Strengthening the Reporting of Observational Studies in Epidemiology—Sports Injury and Illness Surveillance (STROBE-SIIS) [29]. The STROBE-SIIS is a checklist providing guidelines for reporting on surveillance studies for illness and injury in sports. This is a preliminary pilot study and—despite its small sample size—it defines a methodology and shows some important results that encourage research to be carried out on a larger sample size.

### 2.1. Ethics Approval and Consent to Participate

A-FLUTS and IPSS questionnaires were utilised electronically to collect data on the presence, severity, and type of LUTSs and their impact on the athlete’s quality of life. Athletes were recruited from within SINI and a women’s world tour professional cycling team via their individual sports and healthcare practitioners. These athletes were contacted via email with the study link and participant information document attached. The data were collected from consenting athletes via Google Forms, where they were given four weeks to complete the questionnaire (June–July 2023) which best related to their gender identity (A-FLUTS for females and IPSS for males). Participants were sent a ‘Participant Information Document’ before completing the questionnaire to inform them of the aims, content, benefits, and risks of participation in the study, based on which they gave their informed consent. Participant data were anonymised, and ethical approval was received for the study in accordance with declaration of Helsinki (Queen’s University Belfast, Northern Ireland, Faculty Reference Number MHLS 23_77).

### 2.2. Data Analysis

Statistical analysis was carried out by grouping the responses to the A-FLUTS questionnaire and the IPSS questionnaire separately. The response to each question on the questionnaires was given a score based on the individual scoring tool used for each questionnaire. The responses for each question were then plotted as a histogram and line graph to assess if the data were normally distributed. Where the data showed normal distribution, the mean and standard deviation of the data were calculated. Where the data showed a skewed distribution, the median maximum and minimum values were calculated. The data in each questionnaire were also grouped by the athlete’s sport, whether they were explosive/sprint athletes or endurance athletes, and the data were then analysed to determine whether there was a difference in the prevalence or severity of LUTSs between these two groups for each sex.

## 3. Results

A total of one hundred and thirty-seven athletes, both male and female (gender proportions unknown as only contact details were received), from SINI and the women’s elite cycling team were contacted to take part in the study. Of those contacted, twenty-eight athletes (ten males and eighteen females) participated in their respective questionnaires, creating a total response rate of 20.4% across the study. Table 1 shows the demographics of the athletes who responded to the questionnaire, including their gender, relevant medical history, and respective sport.

### 3.1. Male Responses via the IPSS

In total, eleven participants responded to the IPPS questionnaire. One participant was removed as they did not confirm their status as a current elite athlete within SINI. The removed participant who did not meet the inclusion criteria was a road cyclist. Of the ten remaining male athletes, only one was a Paralympic athlete. Eighty percent of participants considered themselves to be endurance athletes, with the other twenty percent referring to themselves as explosive/sprint athletes. The median amount of time spent in each respective sport was 5–10 years. Table 2 shows the median, maximum, and minimum IPSS scores reported by the athletes, both for individual symptoms and total scores. Each symptom was rated on a scale of 0–5, where 0 = ‘Never’ and 5 = ‘Always’. The median total score was 5.5, which falls into the ‘mild symptoms’ category of results [12]. The minimum score obtained was 0, which correlates to no symptoms, and the highest was 35, which correlates with severe symptoms. The individual reporting this score was a boxer. The most commonly experienced LUTS in the IPSS was frequency, with the highest median score at 2 and the highest total score at 24.

The median, maximum, and minimum scores were allocated by the male athletes for UI symptoms on a scale of 0–4, where 0 = ‘Never’ and 4 = ‘Always’. The impact that each of these symptoms had on the quality of life (QOL) of these athletes was also reported on a scale of 0–10, where 0 = ‘None’ and 10 = ‘a great deal’. The median score for all the symptoms was 0. Thus, although the LUTSs were mild, they had little impact on QOL. The highest scoring form of UI was stress UI when coughing or sneezing. This occurred in one athlete whose sport was wheelchair basketball. The symptom receiving the highest score for QOL was UI, receiving a score of 10. Twenty percent of the male athletes experienced UI within the last four weeks. One participant (wheelchair basketball) reported always experiencing UI during match play, competition, strength training, feet conditioning, speed and agility training, and both high and low-intensity running conditioning training, whilst experiencing it most of the time during mobility work and sometimes during plyometric training. Another participant reported only experiencing UI occasionally when performing low-intensity running conditioning. Part of the questionnaire assessed management strategies for those who experience UI. Of the 20% that have experienced UI in training, 100% reported occasionally limiting their fluid intake to reduce the risk of UI, whilst 50% reported always using pads/absorbency products, sometimes changing underwear/training kit, and sometimes modifying the risk of physical exertion to avoid UI. Twenty percent of the male athletes reported that they would be unhappy if their urinary symptoms remained as they were at the time of filling out the questionnaire throughout their lifetime. As previously mentioned, this preliminary study is a pilot model and ideally this study would be repeated on a larger scale to reflect more representative results.

### 3.2. Female Responses via the A-FLUTS

A total of 18 participants completed the A-FLUTS questionnaire, none of which were Paralympic athletes. The participant demographics for female athletes can be seen in Table 1. The median range for time spent as a professional female athlete was 2–5 years. All participants were nulliparous females. A total of 38.9% of participants had a diagnosis of REDS, whilst 33.3% of women who experienced UI also had a diagnosis of RED-S. Table 3 shows the reported total AFLUTS score by the female athletes for several LUTSs, graded on a scale from 0 to 4, where 0 = ‘Never’ and 4 = ‘Always’. The impact that each of these symptoms has on the participant’s QOL was also graded on a scale of 0–10, where 0 = ‘None’ and 10 = ‘a great deal’. The median total A-FLUTS score was reported as 6 out of a total of 44, suggesting the presence of mild LUTSs. The maximum score was 16 and was experienced by a road cyclist. The minimum score was 2, experienced by a cricketer, a triathlete, a rugby player and two road cyclists. The LUTSs occurring whilst undertaking running activities were urinary frequency, hesitancy, nocturia and intermittency. The symptom receiving the greatest median score for impact on QOL was UI. A total of 66.7% of the female athletes reported experiencing UI at least occasionally in the last four weeks.

High-intensity running training, including sprints, received the highest total score for the presence of UI, with a total score of 11. Tactical/technical training had the lowest total score for the presence of UI, with a score of 4.

**Figure 3:**
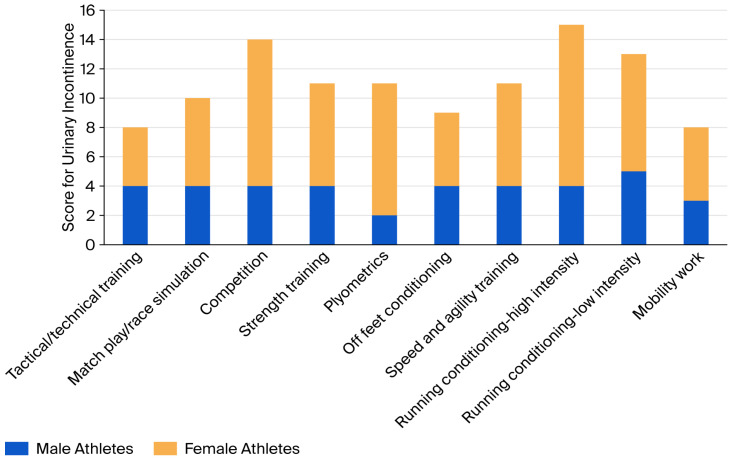
A bar chart comparing the combined total reported scores for UI in each training activity, comparing males to females.

Figure 3 shows a bar chart comparing the combined total reported scores for UI in each training activity, comparing males to females. The scores for males and females cannot be directly compared as there were more female participants in the study. However, the trends between UI in particular activities can be compared. Males and females are not seen to follow the same trends for the activities which cause the greatest UI, with females scoring highest in competition, plyometrics and high-intensity running conditioning and males scoring highest in low-intensity running training.

Table 4 shows the percentages of female athletes who use each method to manage their UI if experienced. Changing underwear/ training kit was the most common management strategy that was reported, with 50% of female athletes using this method at least occasionally.

**Figure 4:**
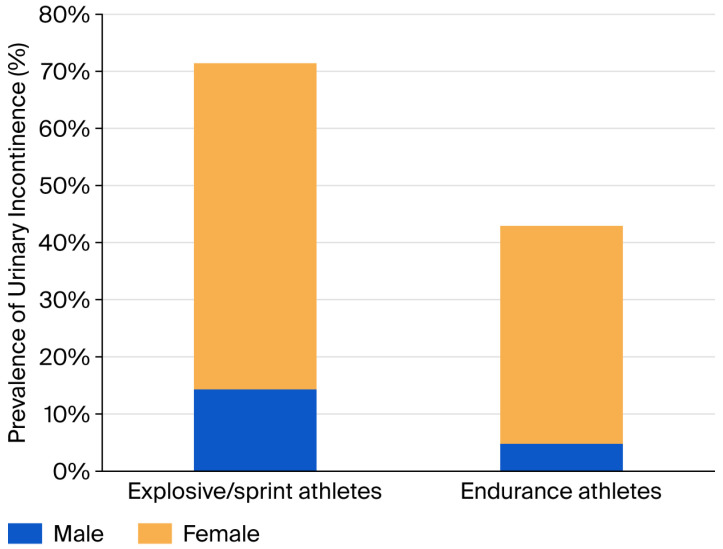
A comparison between the prevalence of UI between explosive/sprint athletes and endurance athletes.

Figure 4 shows a comparison between the prevalence of UI between explosive/sprint athletes and endurance athletes. Across all genders, the study had 21 participants who identified themselves as endurance athletes and 7 explosive/sprint athletes. The median age of the endurance athletes was 26, and the median age of the explosive/sprint athletes was also 26. The prevalence of UI was greatest in explosive/sprint athletes, with 71.4% of these athletes reporting UI at least occasionally over the past four weeks, compared to 42.9% in endurance athletes. This same trend was seen between genders. All of the male athletes who had reported experiencing UI at some point within the last four weeks also reported experiencing UI during training or competition within their respective sport. This suggests that UI may have had some impact on their performance in training or competition. Of the female athletes, only 75% who experienced UI in the last four months also experienced it during training or competition. Further investigation is required following this preliminary study to gain more representative results. The study would need to be carried out with a larger sample size to confirm these findings.

## 4. Discussion

This pilot study aimed to investigate the prevalence of LUTSs (including UI) and their effect on the QOL of elite athletes, investigating male and female athletes separately with their own specially tailored questionnaire. The preliminary study found that the median IPPS score in elite male athletes was 5.5, which correlated with mild urinary symptoms [12]. The median ALFUTS score reported by female elite athletes in this study was 6 out of a possible 44. Female athletes had a higher prevalence of UI in the last four weeks (66.7%) compared to male athletes (20%). However, it is difficult to compare these two groups due to the differences in anatomy and physiology. The prevalence of RED-S in female athletes was 38.9%. There is a greater presence of LUTSs in the athletic population in comparison to the general population of a similar age group [5,30]. Of the 28 athlete participants, 7 were explosive/sprint athletes, and 21 were endurance athletes. Explosive/sprint athletes (71.4%) appeared to have a higher prevalence of UI in the last four weeks compared to endurance athletes (42.9%). The QOL impact of each LUTS was investigated, with UI receiving the highest total scores for effect on QOL in both males and females. As previously mentioned, this is a preliminary study and, although small, shows some important results; most importantly, this study should be expanded to a larger scale. This study will need to be repeated with a larger sample size to confirm the findings.

The authors chose the A-FLUTS questionnaire as it was adapted from the ICIQ, which, in a review of questionnaires, was the most recommended for use in UI and LUTS investigation [31]. It is also adapted to suit athletes, which is particularly suitable for this study. For males, the IPSS was the chosen questionnaire for LUTSs as it is recommended in the NICE guidelines and can be used and compared to studies internationally [29].

There is no official guidance in the literature on the ICIQ FLUTS/A-FLUTS questionnaires to determine the reference ranges for mild, moderate, and severe LUTSs in females. Whilst many studies have used the IPSS and A-FLUTS questionnaires to report on UI in elite athletes, little data could be found on the reporting of specific average scores for each questionnaire type. Therefore, this study provides this unique insight. The highest IPSS score given was the maximum score possible of 35 and was experienced by a male boxer. Interestingly, this individual, whilst scoring the highest for LUTSs in the IPSS, reported no UI. As boxing is considered a high-impact, explosive sport [32], this contradicts previous study conclusions that high-impact sports are associated with a high prevalence of UI and low-impact sports are associated with increased LUTS prevalence [33]. That study focused only on female athletes; therefore, these findings may not be transferable to the male athletic population. However, as this is based on only one participant, it is limited in scientific validity/applicability, and larger studies would need to be completed to provide more conclusive results. Both sexes reported frequency as at least one of the most common LUTSs experienced (in females, hesitancy, nocturia, and intermittency also received the highest median score). This, however, may not be due to athletic factors and could be related to lifestyle factors that were not reported on in our questionnaire, for example, moderate alcohol use or caffeine intake, which has been seen to increase the risk of developing LUTSs [34].

This study reported that the prevalence of UI in elite athletes was 20% in males and 66.7% in female athletes, with these athletes experiencing UI at least occasionally at some point in the four weeks before completing the questionnaire. This, therefore, gave a 50% prevalence of UI in elite athletes of all genders. Additionally, these results also mirror previous study findings that female athletes are more likely than male athletes to experience UI [7]. Due to the limited number of participants involved, the results of this preliminary survey remain inconclusive regarding the possibility of a higher prevalence of LUTSs in athletes compared to the general population. The authors’ aim would be to complete this study on a larger scale to gain more conclusive evidence and compare how this correlates with this study’s findings. This study provides the necessary methodology and framework for this area to be further researched.

The median age of the male athletic population was 23 compared to 26 in the female athlete population, and the median BMI in the male athlete population was 20.8, compared with 21.6 in the female population. Therefore, the female athletic population had a slightly increased median age and BMI compared to the male athletic population. UI prevalence has been seen to increase with increasing age and BMI [35,36,37]. It is, therefore, possible that these factors contributed slightly to the results of increased prevalence of UI in female athletes compared to male athletes.

Whilst there is limited research into UI prevalence in elite male athletes, this study’s value of 20% is greater than that reported in a previous study. Indeed, Rodríguez-López et al. reported a UI prevalence of 14.7% in male athletes [7]. This may be due to the small sample size of the male participant population. Moreover, the reported UI prevalence of 66.7% in female athletes was greater than that reported in previous research, suggesting that the prevalence of UI in young female athletes was, on average, 44% [4].

The investigation into how these symptoms affected the athletes’ QOL determined that the most bothersome LUTS for both males and females was UI. In female athletes, the management strategies used can be seen in Table 4. These results further highlight the QOL impact of UI and how bothersome the management of the symptom can be. Changing kits, underwear or pads/absorbency products regularly can be expensive and time-consuming, taking time out of training, which could potentially impact performance. Furthermore, pads/absorbency products and extra kits/washing are an added financial expense for these athletes. Both male and female athletes reported decreasing their level of exertion to minimise the risk of experiencing UI, which once again could greatly impact their performance in their respective sports by reducing their training volumes.

Studies suggest that water intake in elite endurance athletes is usually inadequate [38]. Therefore, if athletes are consciously attempting to limit their water intake to minimise the risk of experiencing UI, this could affect not only their sporting performance but also their general health as it could reduce their hydration status and electrolyte balance [38]. Therefore, the performance team need to address UI in their assessment of athletes, e.g., in the pre-season screening using a recognised, valid questionnaire, such as the International Olympic Committee (IOC) preparticipation questionnaire, which should be updated to include urinary symptoms [39]. Previous studies show that 20% of women left their respective sport due to the QOL impacts of UI, highlighting its significance [25]. The authors reported a 38.9% prevalence of RED-S in female athletes, which is less than the 41.2% prevalence of RED-S reported in female elite athletes from Malaysia [40]. No information on race or ethnicity was obtained from this study’s female athlete sample.

This study investigated the differences in the prevalence of UI between explosive/sprint and endurance athletes. Explosive/sprint athletes experienced a UI prevalence of 71.4%, whilst endurance athletes experienced a significantly lower UI prevalence of 42.9%. The median ages of both endurance and explosive/sprint athletes were 26. The trend of explosive/sprint athletes experiencing a greater prevalence of UI compared to endurance athletes was also supported when the results were split and analysed for both males and females. Interestingly, there are gaps in the literature on the difference in prevalence of UI between explosive/sprint athletes and endurance athletes. Thus, this study provides a unique insight into this topic, although more research would need to be carried out in this area following this preliminary study to confirm its findings. This study provides the necessary framework for this research to be completed on a larger scale and compared with the present results. Although no specific papers on LUTSs in endurance compared to explosive/sprint athletes could be found, there is existing evidence of an increased prevalence of UI in high-impact sports compared to low-impact sports [35]. Explosive/sprint athletes often demonstrate small bursts of explosive athletic activity, which is associated with high-impact activity. It is this high-impact activity that is thought to increase intra-abdominal pressure either against the action of previously weakened pelvic floor muscles or causing damage to the pelvic floor muscles attempting to withstand the pressure [41]. This is potentially why explosive/sprint athletes have a greater prevalence of UI than endurance athletes, who participate in more low-impact activities. Although endurance athletes are at a higher risk of RED-S, the prevalence in this study was 42.9% in endurance athletes and 71.4% in explosive sports [28].

### Study Strengths and Limitations

The pilot study was conducted following the STROBE-SIIS checklist to ensure that the questionnaire was appropriate, and that all necessary data were gathered for completing the study. The PROMs (both AFLUTS and IPSS) were useful tools that allowed data to be collected remotely and anonymously whilst being user-friendly and not very time-consuming to complete, only taking a few minutes [7]. The IPSS questionnaire takes 165 s on average to complete [42]. This may have contributed to the positive response rate of 20.4% in this study. Response rates within primary care studies have been reported to vary between 10.3% and 61% [43]. Whilst the response rate is on the smaller end of this range, it can still be considered a strength of this study as it still falls within this range despite focusing on topics which may be considered embarrassing to participants. The use of the IPPS and A-FLUTS questionnaires provided thorough data collection from participants, with questions specifically aimed at their respective target audience. The IPPS questionnaire has been validated and is recommended by NICE in the United Kingdom [8]. Similarly, the ICIQ FLUTS questionnaire from which A-FLUTS has been adapted has established validity, reliability, and responsiveness [11,44].

Potential limitations of this study includes the study sample size, which was relatively small, especially in terms of male athletes. One response had to be discounted due to the participant not meeting the inclusion criteria. Unfortunately, due to the small sample size, statistical tests could not be carried out. This is a preliminary pilot study and—despite its small size—it defines a methodology and shows some important results that encourage research to be carried out on a larger sample size. The authors’ goal was to provide this necessary research foundation so that the methodology can be utilised on a larger sample size and the results compared. Typically, in research, the prevalence of LUTSs is underrepresented due to potential embarrassment related to the topic, which could potentially cause the results of this study to be an underrepresentation of LUTS prevalence [4]. With IPSS, the prevalence of LUTSs is calculated as a summed score (0 = none, 1–7 = mild, 8–19 = moderate and 19–35= severe). This may have limited the study as it does not report on individual symptoms and therefore limits the ability to consider the prevalence of individual LUTSs and the impact that each has on patients’ quality of life [3]. While the use of the AFLUTS questionnaire added strength to this study, the removal of one question due to a technical problem limited the ability to fully compare results directly with other studies, making it more difficult to compare the elite female athlete population with other similar populations. Another limitation of this study was the lack of information received on other risk factors of LUTSs and UI. While information on RED-S diagnosis, age and obstetric history was obtained, information on other factors that could have contributed to LUTS and UI prevalence, including past medical history, medication history, caffeine intake, hormonal status, and surgical history, was not obtained. Further work assessing the prevalence of urinary symptoms in the athletic cohort is therefore required.

## 5. Conclusions

In conclusion, whilst the prevalence of LUTSs and UI in female athletes has been reported relatively frequently in the literature, it is important to recognise that male athletes also suffer from these symptoms. This primary pilot study highlighted the potentially significant impact that these symptoms have on athletes, including effects on their physical health, mental health, and athletic performance, particularly in terms of reducing fluid intake. It is, therefore, crucial that healthcare providers become more aware of the prevalence of these symptoms in elite athletes, screen for them and then develop appropriate treatment pathways to achieve the best management and treatment outcomes in this population. This pilot study underscores the importance of further research in this area and for research to be conducted on a larger scale with a larger sample size. This study suggests the need to screen for urinary symptoms on an annual basis within athletic populations in the pre-season medical as well as for UI risk factors to ensure a holistic approach to athlete health.

## Figures and Tables

**Table 1:** A table showing athlete demographics and breakdown via sport.

		Males				Females			
Demographic Data		Mean	SD	Median	Range	Mean	SD	Median	Range
Age (years)		-	-	23	17–38	-	-	26	17–34
Weight (kg)		-	-	70	48–76	-	-	63.4	50.5–83
Height (m)		173.1	12.8	-	-	166.5	5.15	-	-
BMI (kg/m^2^)		-	-	20.8	16.6–23.7	-	-	21.6	18.5–29.3
Medical History		n			%	n			%
Constipation		0			0	1			5.6
Frequent urinary tract infections		1			10	3			16.7
Family history of urinary incontinence		1			10	0			0
Relative Energy Deficiency in Sport (RED-S)		0			0	7			38.9
Pregnancies		-			-	0			0
Sport		n			%	n			%
Endurance Athlete		8			80	13			72.2
-Cycling	Road	1			10	11			61.1
-Swimming	Breaststroke	2			20	-			-
-Bowls	Team	1			10	-			-
-Rugby		-			-	1			5.6
-Triathlon	Sprint distance	2			20	1			5.6
-Boxing	Weight cut	1			10	-			-
-Athletics	Mountain running	1			10	-			-
Explosive/sprint athlete		2			20	5			27.8
-Cricket	Bowling	-			-	3			16.7
	Batting	-			-	0			0
-Judo		-			-	1			5.6
-Cycling	Road	-			-	1			5.6
-Boxing	Consistency	1			10	-			-
-Basketball	Wheelchair	1			10	-			-

**Table 2:** The median, maximum, and minimum IPSS scores reported by the male athletes, both for individual symptoms and total scores.

Symptom	Median Score	Range
Incomplete emptying	0.5	0–5
Frequency	2	0–5
Intermittency	0	0–5
Urgency	0	0–5
Weak stream	0	0–5
Straining	0	0–5
Nocturia	1	0–5
Total IPSS score	5.5	0–35

**Table 3:** Reported total AFLUTS score by the female athletes for several lower urinary tract symptoms (LUTSs), graded on a scale from 0 to 4, where 0 = ‘Never’ and 4 = ‘Always’.

Symptom	Median Score (/4)	Score Range (/4)	Median Quality of Life (QOL) Impact (/10)	QOL Impact Range
Nocturia	1	0–3	2	0–8
Urgency	0.5	0–3	1	0–8
Bladder pain	0	0–2	0	0–5
Frequency	1	0–4	1	0–8
Hesitancy	1	0–2	0	0–4
Straining	0	0–2	0	0–3
Intermittency	1	0–2	0	0–3
Urinary Incontinence (UI)	0	0–3	0	0–9
UI without warning	0	0–2	0	0–8
UI when asleep	0	0–1	0	0–3
UI on coughing or sneezing	0	0–2	0	0–8
Total A-FLUTS score (/44)	6	2–16		

**Table 4:** Percentages of female athletes using specific strategies to manage urinary incontinence.

	Never	Occasionally	Sometimes	Most of the Time	Always
Management of urinary incontinence (UI) if experienced (n = 18)					
Pads/absorbency products	88.9%	5.6%	-	5.6%	-
Change underwear/training kit regularly	50%	22.2%	16.7%	11.1%	-
Restrict fluid intake	77.8%	11.1%	11.1%	-	-
Modify level of exertion	88.9%	5.6%	5.6%	-	-
Medication	100%	-	-	-	-

## Data Availability

The original contributions presented in this study are included in the article. Further inquiries can be directed to the corresponding authors.

## Abbreviations

The following abbreviations are used in this manuscript:

A-FLUTS	Athlete Female Lower Urinary Tract Symptoms
ICIQ	International Consultation on Incontinence Questionnaires
IPPS	International Prostate Symptom Score
LUTS	Lower Urinary Tract Symptom
NICE	National Institute for Health and Care Excellence
PROMs	Patient-Reported Outcome Measures
RED-S	Relative Energy Deficiency in Sport
SIJ	Sacroiliac Joint
UI	Urinary Incontinence
UK	United Kingdom
SINI	Sport Institute of Northern Ireland

## References

[1] Przydacz M., Gasowski J., Grodzicki T., Chlosta P. (2023). Lower Urinary Tract Symptoms and Overactive Bladder in a Large Cohort of Older Poles—A Representative Tele-Survey. J. Clin. Med..

[2] Beland L., Martin C., Han J.S. (2022). Lower Urinary Tract Symptoms in Young Men—Causes and Management. Curr. Urol. Rep..

[3] Coyne K.S., Sexton C.C., Thompson C.L., Milsom I., Irwin D., Kopp Z.S., Chapple C.R., Kaplan S., Tubaro A., Aiyer L.P. (2009). The prevalence of lower urinary tract symptoms (LUTS) in the USA, the UK and Sweden: Results from the Epidemiology of LUTS (EpiLUTS) study. BJU Int..

[4] Casey E.K., Temme K. (2017). Pelvic floor muscle function and urinary incontinence in the female athlete. Physician Sportsmed..

[5] Teixeira R.V., Colla C., Sbruzzi G., Mallmann A., Paiva L.L. (2018). Prevalence of urinary incontinence in female athletes: A systematic review with meta-analysis. Int. Urogynecol. J..

[6] Almousa S., Bandin Van Loon A. (2019). The prevalence of urinary incontinence in nulliparous female sportswomen: A systematic review. J. Sports Sci..

[7] Rodríguez-López E.S., Calvo-Moreno S.O., Basas-García Á., Gutierrez-Ortega F., Guodemar-Pérez J., Acevedo-Gómez M.B. (2021). Prevalence of urinary incontinence among elite athletes of both sexes. J. Sci. Med. Sport.

[8] Rees J. (2010). NICE guideline on lower urinary tract symptoms in men. Trends Urol. Men’s Health.

[9] NICE (2019). NICE Guidance—Urinary incontinence and pelvic organ prolapse in women: Management. BJU Int..

[10] Uren A.D., Cotterill N., Pardoe M., Abrams P. (2020). The International Consultation on Incontinence Questionnaires (ICIQ): An update on status and direction. Neurourol. Urodyn..

[11] Jackson S., Donovan J., Brookes S., Eckford S., Swithinbank L., Abrams P. (1996). The Bristol Female Lower Urinary Tract Symptoms questionnaire: Development and psychometric testing. BJU Int..

[12] Barry M.J., Fowler F.J., O’leary M.P., Bruskewitz R.C., Holtgrewe H.L., Mebust W.K., Cockett A.T., The Measurement Committee of the American Urological Association (1992). The American Urological Association Symptom Index for Benign Prostatic Hyperplasia. J. Urol..

[13] Vleeming A., Schuenke M.D., Masi A.T., Carreiro J.E., Danneels L., Willard F.H. (2012). The sacroiliac joint: An overview of its anatomy, function and potential clinical implications. J. Anat..

[14] Bø K., Sherburn M. (2005). Evaluation of female pelvic-floor muscle function and strength. Phys. Ther..

[15] Prather H., Dugan S., Fitzgerald C., Hunt D. (2009). Review of Anatomy, Evaluation, and Treatment of Musculoskeletal Pelvic Floor Pain in Women. PM&R.

[16] Wester C., Brubaker L. (1998). Normal Pelvic Floor Physiology. Obstet. Gynecol. Clin. N. Am..

[17] Kennedy J. (2008). Neurologic injuries in cycling and bike riding. Neurol. Clin..

[18] Delaney T., Young D.C. (2003). Spontaneous versus induced labor after a previous cesarean delivery. Obstet. Gynecol..

[19] Seike K., Koda K., Oda K., Kosugi C., Shimizu K., Miyazaki M. (2009). Gender differences in pelvic anatomy and effects on rectal cancer surgery. Hepatogastroenterology.

[20] DeSilva J.M., Rosenberg K.R. (2017). Anatomy, Development, and Function of the Human Pelvis. Anat. Rec..

[21] Wu Y., Hikspoors J.P., Mommen G., Dabhoiwala N.F., Hu X., Tan L., Zhang S., Lamers W.H. (2020). Interactive three-dimensional teaching models of the female and male pelvic floor. Clin. Anat..

[22] Yucel S., Baskin L.S. (2004). An Anatomical Description of the Male and Female Urethral Sphincter Complex. J. Urol..

[23] Stoker J., Wallner C. (2008). The Anatomy of the Pelvic Floor and Sphincters. Imaging Pelvic Floor Disorders.

[24] Gowda S.N., Bordoni B. (2023). Anatomy, Abdomen and Pelvis: Levator Ani Muscle. StatPearls.

[25] Carvalhais A., Natal Jorge R., Bø K. (2018). Performing high-level sport is strongly associated with urinary incontinence in elite athletes: A comparative study of 372 elite female athletes and 372 controls. Br. J. Sports Med..

[26] Holroyd-Leduc J.M., Tannenbaum C., Thorpe K.E., Straus S.E. (2008). What Type Urin. Incontinence Does This Woman Have?. JAMA.

[27] Borin L.C.M.d.S., Nunes F.R., Guirro E.C.d.O. (2013). Assessment of Pelvic Floor Muscle Pressure in Female Athletes. PM&R.

[28] Rebullido T.R., Stracciolini A. (2019). Pelvic Floor Dysfunction in Female Athletes: Is Relative Energy Deficiency in Sport a Risk Factor?. Curr. Sports Med. Rep..

[29] Bahr R., Clarsen B., Derman W., Dvorak J., Emery C.A., Finch C.F., Hägglund M., Junge A., Kemp S., Khan K.M. (2020). International Olympic Committee consensus statement: Methods for recording and reporting of epidemiological data on injury and illness in sport 2020 (including STROBE Extension for Sport Injury and Illness Surveillance (STROBE-SIIS)). Br. J. Sports Med..

[30] Thom D. (1998). Variation in Estimates of Urinary Incontinence Prevalence in the Community: Effects of Differences in Definition, Population Characteristics, and Study Type. J. Am. Geriatr. Soc..

[31] Avery K., Bosch J., Gotoh M., Naughton M., Jackson S., Radley S., Valiquette L., Batista J., Donovan J. (2007). Questionnaires to Assess Urinary and Anal Incontinence: Review and Recommendations. J. Urol..

[32] Ruddock A.D., Wilson D.C., Thompson S.W., Hembrough D., Winter E.M. (2016). Strength and Conditioning for Professional Boxing. Strength Cond. J..

[33] Simeone C., Moroni A., Pettenò A., Antonelli A., Zani D., Orizio C., Cunico S.C. (2010). Occurrence Rates and Predictors of Lower Urinary Tract Symptoms and Incontinence in Female Athletes. Urol. J..

[34] Bradley C.S., Erickson B.A., Messersmith E.E., Pelletier-Cameron A., Lai H.H., Kreder K.J., Yang C.C., Merion R.M., Bavendam T.G., Kirkali Z. (2017). Evidence of the Impact of Diet, Fluid Intake, Caffeine, Alcohol and Tobacco on Lower Urinary Tract Symptoms: A Systematic Review. J. Urol..

[35] Lourenco T.R.d.M., Matsuoka P.K., Baracat E.C., Haddad J.M. (2018). Urinary incontinence in female athletes: A systematic review. Int. Urogynecol. J..

[36] Parazzini F. (2003). Risk factors for stress, urge or mixed urinary incontinence in Italy. BJOG.

[37] Shamliyan T.A., Wyman J.F., Ping R., Wilt T.J., Kane R.L. (2009). Male urinary incontinence: Prevalence, risk factors, and preventive interventions. Rev. Urol..

[38] Pašalić A., Jusupovic F., Rudić A., Mahmutović J., Branković S., Jaganjac A. (2015). Habits of fluid and electrolytes intake in elite athletes. J. Health Sci..

[39] International Olympic Committee (2009). The IOC Consensus Statement on Periodic Health Evaluation of Elite Athletes. https://olympics.com/ioc/news/the-ioc-consensus-statement-on-periodic-health-evaluation-of-elite-athletes.

[40] Marzuki M.I.H., Mohamad M.I., Chai W.J., Farah N.M.F., Safii N.S., Jasme J.K., Jamil N.A. (2023). Prevalence of Relative Energy Deficiency in Sport (RED-S) among National Athletes in Malaysia. Nutrients.

[41] Pires T., Pires P., Moreira H., Viana R. (2020). Prevalence of Urinary Incontinence in High-Impact Sport Athletes: A Systematic Review and Meta-Analysis. J. Hum. Kinet..

[42] Els M., Heyns C., van der Merwe A., Zarrabi A. (2019). Prospective comparison of the novel visual prostate symptom score (VPSS) versus the international prostate symptom score (IPSS), and assessment of patient pain perception with regard to transrectal ultrasound guided prostate biopsy. Int. Braz. J. Urol..

[43] Booker Q.S., Austin J.D., Balasubramanian B.A. (2021). Survey strategies to increase participant response rates in primary care research studies. Fam. Pract..

[44] Avery K., Donovan J., Peters T.J., Shaw C., Gotoh M., Abrams P. (2004). ICIQ: A brief and robust measure for evaluating the symptoms and impact of urinary incontinence. Neurourol. Urodyn..

